# Supplementary material for: Interface Interactions in Conjugated Polymer Composite with Metal Oxide Nanoparticles
Source: Nanomaterials (Basel). 2019 Oct 29;9(11):1534. doi: 10.3390/nano9111534 (PMC6915360; doi:10.3390/nano9111534)
Supplement: Supplementary file 1 [file nanomaterials-09-01534-s001.pdf]

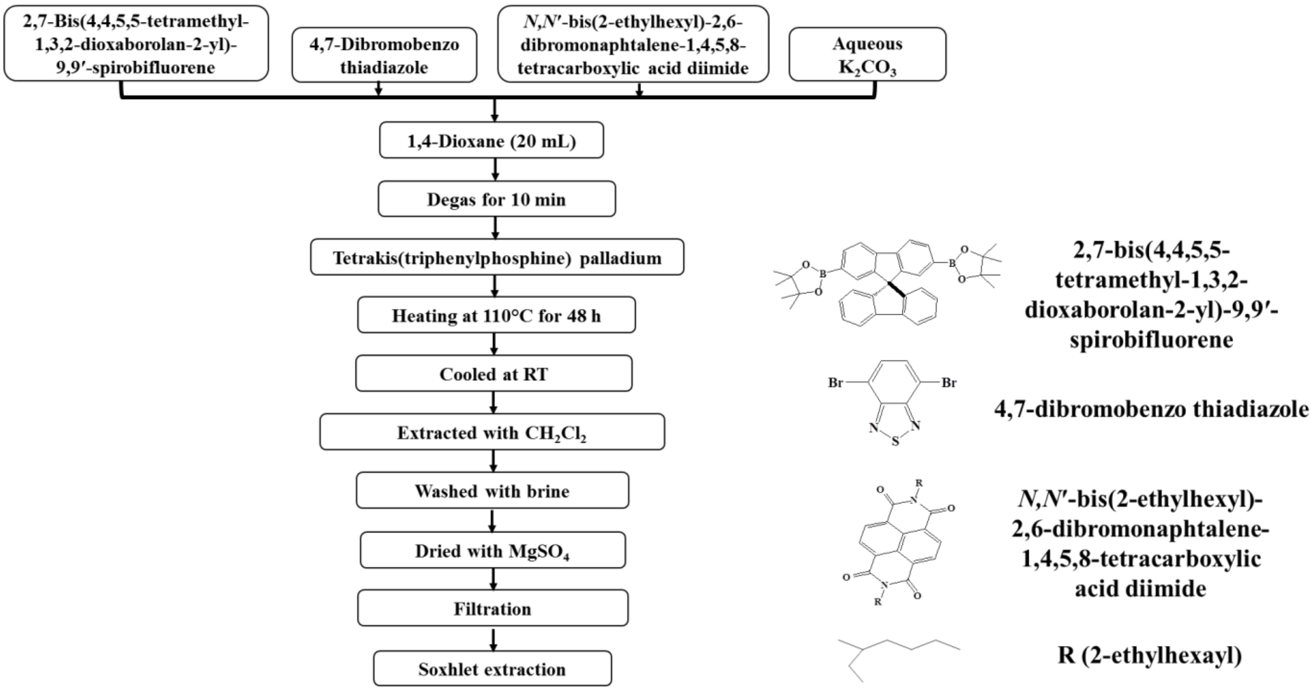

Figure S1 The schematic picture of the polymer synthesis procedure. The chemical formulas of polymers are also shown .

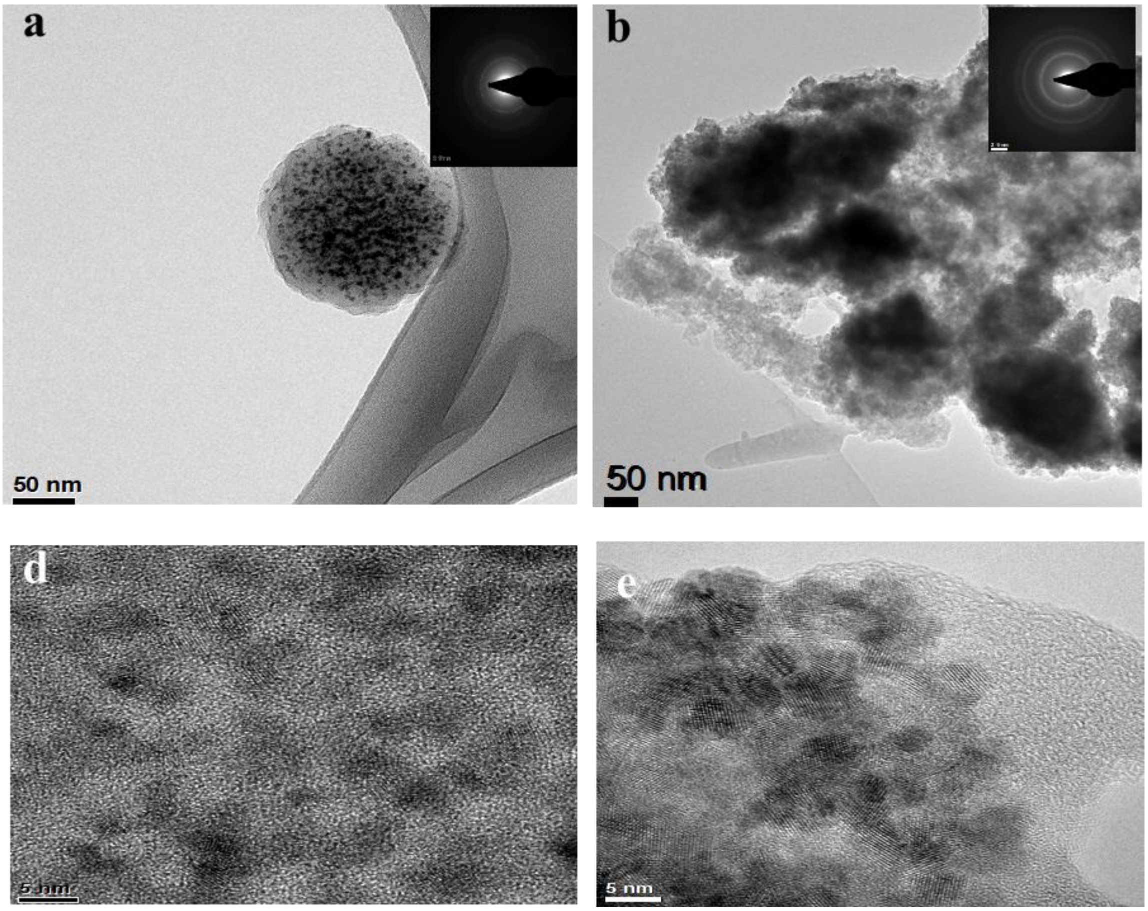

Figure S2 TEM investigation of polymer composite with different amount of CeO_2, (a) bright image of composite with 20wt% CeO_2, (b) bright image of composite with 50wt% CeO_2, (c) HRTEM of composite with 20wt% CeO_2, (d) HRTEM of composite with 50wt% CeO_2. The insert figures were corresponding diffraction patterns. Aggregation of nanoparticles was observed in composite with 50wt% CeO_2.
